# The Emergence of a CRISPR‐Cas Revolution in Ecology: Applications, Challenges, and an Ecologist's Overview of the Toolbox

**DOI:** 10.1111/1755-0998.70086

**Published:** 2025-12-02

**Authors:** Amadeus Plewnia, Brandon D. Hoenig, Stefan Lötters, Christopher Heine, Jesse Erens, Philipp Böning, Gary D. Bending, Henrik Krehenwinkel, Molly Ann Williams

**Affiliations:** ^1^ Department of Biogeography Trier University Trier Germany; ^2^ Department of Biological Sciences University of Pittsburgh Pittsburgh Pennsylvania USA; ^3^ Department of Geobotany Trier University Trier Germany; ^4^ School of Life Sciences University of Warwick Coventry UK

**Keywords:** biodiversity monitoring, CRISPR sequencing, CRISPR‐Dx, environmental DNA, genome editing, nucleic acid detection

## Abstract

CRISPR‐Cas (Clustered Regularly Interspaced Short Palindromic Repeats—CRISPR‐associated nucleases) systems allow researchers to detect, capture, and even alter parts of an organism's genome. However, while the use of CRISPR‐Cas has revolutionised many fields in the life sciences, its full potential remains underutilised in ecology and biodiversity research. Here we outline the emerging applications of CRISPR‐Cas in ecological contexts, focusing on three main areas: nucleic acid detection, CRISPR‐enhanced sequencing, and genome editing. CRISPR‐based nucleic acid detection of environmental DNA samples is already reshaping species monitoring, providing highly sensitive and non‐invasive tools for both scientists and the public alike, with reduced costs and minimal experience required. Further, CRISPR‐enhanced sequencing, including Cas‐mediated target enrichment, enables efficient recovery of ecologically relevant loci and supports diverse applications such as amplification‐free metagenomics. Finally, while genome editing on wild species remains largely theoretical in ecology, these tools are already being used in controlled settings to study adaptation and resilience in the face of ongoing global stressors. Together, the applications of CRISPR‐Cas are paving the way for more affordable, accessible, and impactful applications for species conservation, and promise to improve our ability to tackle the ongoing global biodiversity crisis.

## 1 Introduction

The accelerating pace of global biodiversity loss poses an unprecedented challenge of the Anthropocene (Barnosky et al. 2011). In response to this biodiversity crisis, the Kunming–Montreal Global Biodiversity Framework (GBF) adopted at COP15 aims to protect and restore at least 30% of the world's terrestrial and marine areas while maintaining the genetic diversity within populations to safeguard their adaptive potential across ecosystems (Convention on Biological Diversity 2022). To address this societal challenge, scientists and the public alike must capitalise on novel methodological advancements that allow individuals to more accurately and rapidly characterise, monitor, and protect global biodiversity.

Recent developments—particularly molecular approaches such as environmental DNA (eDNA) and the miniaturisation of field laboratories—can help us work towards these goals (Altermatt et al. 2025). Simultaneously, CRISPR‐Cas (Clustered Regularly Interspaced Short Palindromic Repeats—CRISPR‐associated nucleases) systems have revolutionised the molecular life sciences by allowing researchers to detect, remove, and even alter entire regions of an individual's genetic code (Wang and Doudna 2023). Originally derived from the adaptive immune system of prokaryotes (Horvath and Barrangou 2010), CRISPR‐Cas is today among scientists' most powerful tools to control genetic and vector‐borne disease and has transformed how we approach drug development and agricultural advancement (Hille et al. 2018). Although most known for genome editing, CRISPR‐Cas systems also have broad potential to improve upon widely used molecular approaches, particularly nucleic acid detection (Gootenberg et al. 2017, 2018; Chen et al. 2018) and targeted high‐throughput sequencing (Schultzhaus et al. 2021). However, while CRISPR‐Cas technology is already becoming a cornerstone in organismal, biomedical, and biotechnological research, it appears as though ecologists and conservationists have been slow in utilising the CRISPR‐Cas revolution (Westra et al. 2016) with relatively few research endeavours employing these approaches until recently (Phelps 2019; Phelps et al. 2019; Williams et al. 2019; Baerwald et al. 2020, 2025; Hoenig et al. 2023; Blasko et al. 2024; Blasko and Phelps 2024).

**FIGURE 1:**
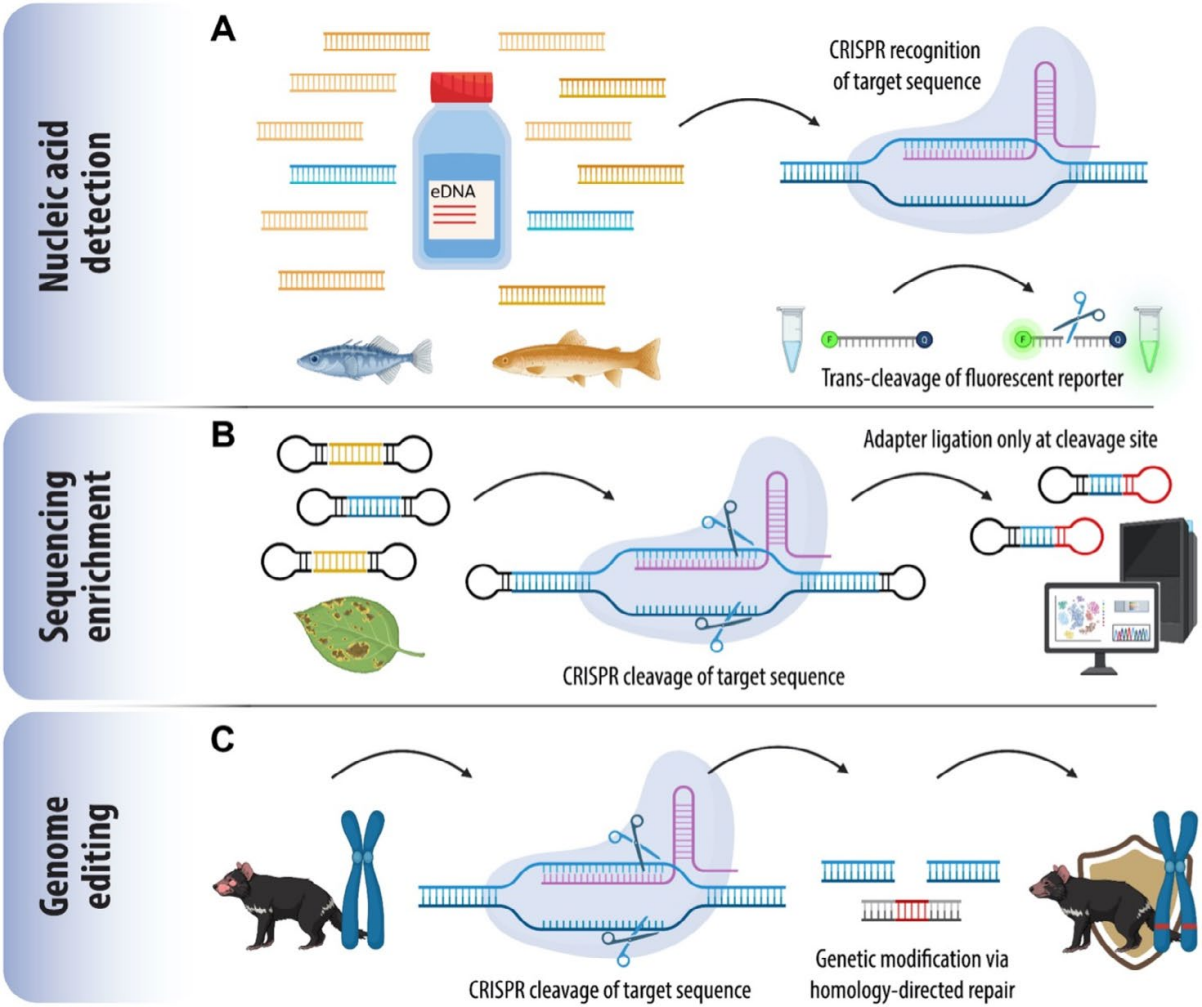
The ecologist's CRISPR‐Cas toolkit. (A) CRISPR‐Cas‐mediated nucleic acid detection. Cas enzymes are activated by the presence of a target sequence, inducing cleavage of fluorescence‐labelled molecules. This enables ultrasensitive detection, for example, for tracing threatened species from environmental samples. (B) CRISPR‐Cas‐mediated sequencing, via targeted cleavage and affinity tag ligation, enriches libraries for high‐throughput sequencing, for example, for detection of active immune genes in response to environmental stressors. (C) Genome editing through homology‐directed repair of cleaved DNA enables genetic modifications, for example, enhancing disease resilience in threatened species or identifying the genomic basis of adaptation. Target DNA displayed in blue, non‐target DNA orange, ribonucleoprotein complex with gRNA pink and Cas nuclease light blue. F indicates fluorophore marker and Q indicates fluorescence quencher. Scissors indicate cleavage. Created using BioRender.

CRISPR‐Cas technology yields highly specific nuclease activity by taking advantage of complementary base pairing between short, synthetic guide RNAs (gRNA) and the target nucleic acid of interest (Gootenberg et al. 2018; Hille et al. 2018). In brief, a programmable gRNA and Cas nuclease form a complex that can recognise a nucleic acid sequence with 17–28 bp complementarity and induce cleavage within this target molecule (Horvath and Barrangou 2010; Kellner et al. 2019). In prokaryotic immunity, this system enables recognition and cleavage of foreign nucleic acids, in turn disabling invasive elements (Garneau et al. 2010). In biotechnology, however, cleavage events can be harnessed by cellular repair mechanisms to modify, delete, or insert new genetic material at the break site (Anzalone et al. 2020; Wang and Doudna 2023; Figure 1C). For a detailed review on CRISPR‐Cas technology refer to Wang and Doudna (2023). Beyond genome editing, the identification, characterisation, and modification of novel Cas nucleases has enabled CRISPR‐Cas systems to be used for other applications (Barrangou and Doudna 2016). For example, some Cas nucleases (e.g., Cas12 or Cas13) demonstrate indiscriminate cleavage of single‐stranded nucleic acids once they have recognised their intended target sequence, a property known as trans cleavage activity. This activity can be redirected towards synthetic reporter molecules (e.g., short fluorescent or affinity‐tagged probes), so that their degradation provides an indirect but highly sensitive signal for nuclease activation and hence the presence of the target nucleic acid (Gootenberg et al. 2017, 2018; Chen et al. 2018; Kellner et al. 2019; Abudayyeh and Gootenberg 2021; Figure 1A). This method is commonly known as CRISPR diagnostics or CRISPR‐Dx. It can often be used on‐site and without specialised equipment due to the ability of certain Cas enzymes to function in less than 1 h, for instance using miniaturised incubation devices or body heat with subsequent lateral flow readout (Gootenberg et al. 2018). Further, CRISPR‐Cas applications have been broadened by modified nucleases such as the catalytically inactive nuclease dCas9 for control of gene expression (Qi et al. 2013) or the more thermostable nuclease eBrCas12b for integration with DNA amplification (Nguyen et al. 2023). Additionally, site‐directed CRISPR‐Cas cleavage can aid in the preparation of targeted reduced representation libraries for high‐throughput sequencing, while modified nuclease‐deficient Cas enzymes enable more precise capture of genomic fragments (Schultzhaus et al. 2021; Ramón‐Laca et al. 2023; Figure 1B).

Contrasting ‘traditional’ molecular techniques like quantitative PCR or high‐throughput sequencing, CRISPR‐Cas technology is remarkably cost‐efficient and field‐deployable, democratising and enabling simplified real‐time investigation without requiring extensive infrastructure or laboratory experience (Abudayyeh and Gootenberg 2021). As such, CRISPR‐Cas‐based nucleic acid detection is already experiencing a significant surge in biodiversity monitoring applications (termed CRISPR‐eBx by Durán‐Vinet et al. 2025), spanning applications in pathogen detection, species monitoring, and genotyping or identification of cryptic species, all of which can be applied to environmental samples or specimens (Phelps 2019; Phelps et al. 2019; Williams et al. 2019, 2021, 2023; Baerwald et al. 2020, 2025; Hoenig et al. 2023, 2024; Blasko et al. 2024; Blasko and Phelps 2024).

This mini review outlines the recent progress in ecological CRISPR‐Cas applications, highlighting key areas of interest where CRISPR‐Cas technology could overcome current methodological limitations, and identifying knowledge gaps and high‐priority research areas to fully harness the CRISPR‐Cas revolution in ecology.

## 2 Applications of CRISPR‐Cas in Ecology and Biodiversity Research

To date, ecological applications of CRISPR‐Cas systems have ranged from assisting gene flow and engineering disease resistance in vulnerable populations to identifying gene function in evolutionary ecology studies (Bono et al. 2015; Phelps 2019; Yin et al. 2024). However, the application that has already shown the most promise in conservation ecology is simplified and cost‐efficient on‐site nucleic acid detection. Due to its versatility in biodiversity monitoring, CRISPR‐Cas systems have been coupled with eDNA techniques for non‐invasive species detection (Williams et al. 2019), have rapidly distinguished migration phenotypes of anadromous fish based on minimally invasive samples (Baerwald et al. 2025), and have unravelled pine tree‐nematode interactions in real‐time (Wang et al. 2022).

### 2.1 Nucleic Acid Detection

**FIGURE 2:**
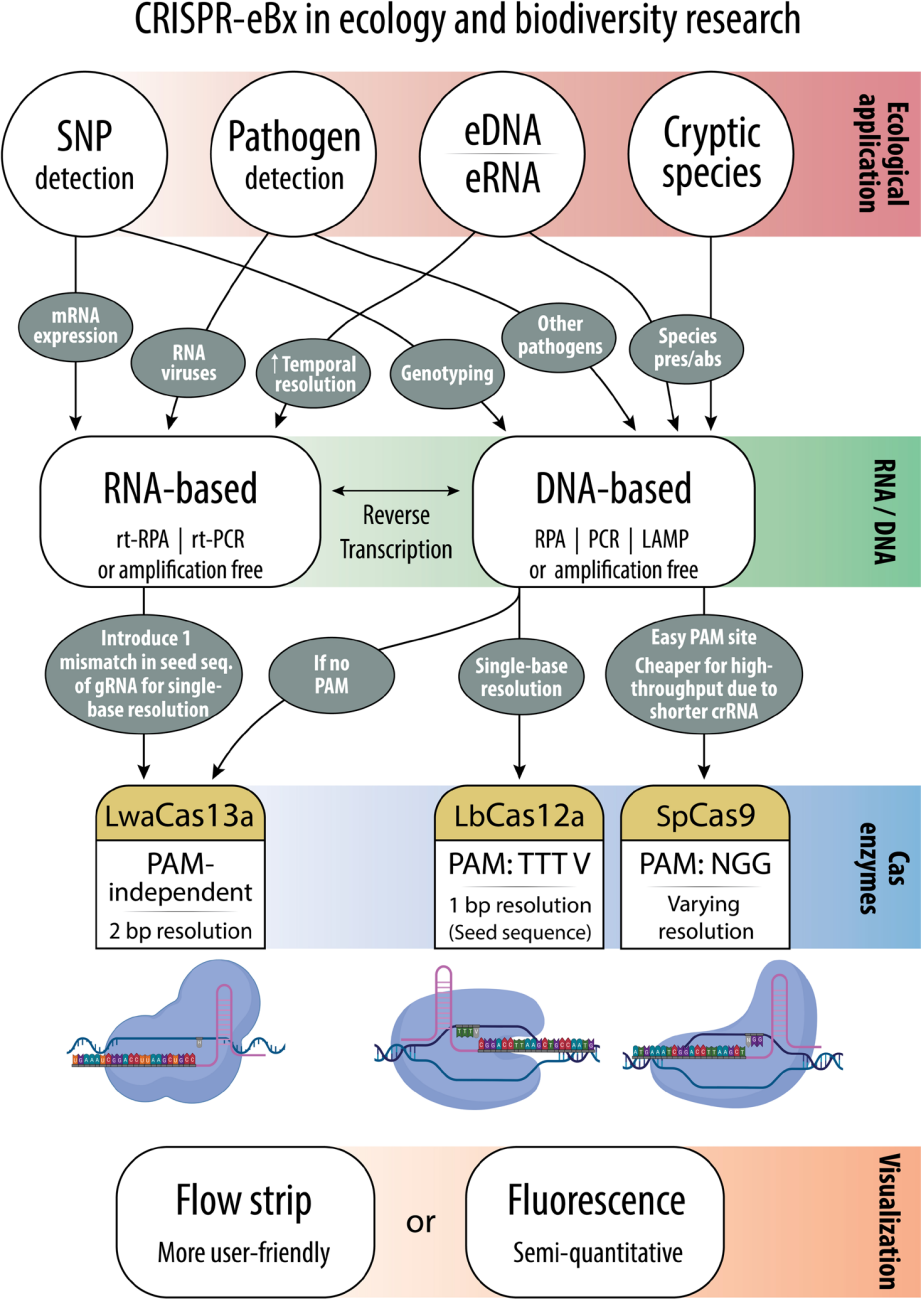
An overview of the potential uses and considerations for the CRISPR‐eBx toolbox in ecology and biodiversity science. Note that seed sequence specificity and PAM architecture may vary between different bacterial lineages employed for Cas production.

The majority of CRISPR‐Cas studies in the field of ecology have focused on its use in nucleic acid detection (herein referred to as CRISPR‐eBx, Durán‐Vinet et al. 2025) from specimen swabs and eDNA samples. To date, CRISPR‐eBx protocols are amplification‐based using either traditional PCR (Leugger et al. 2025; Osathanunkul 2025; Zhang et al. 2024) or more field‐deployable isothermal techniques such as Recombinase Polymerase Amplification (RPA) (Williams et al. 2019, 2021, 2023; Baerwald et al. 2020, 2025; Hoenig et al. 2023, 2024) and Loop‐mediated Isothermal Amplification (LAMP) (Blasko et al. 2024; Blasko and Phelps 2024). When using off‐the‐shelf Cas nucleases, both PCR and LAMP require a two‐pot reaction due to the need for incubation temperatures outside of the Cas range. RPA, on the other hand, can be either run as a two‐pot reaction or combined with CRISPR‐Cas detection into a one‐pot reaction as both steps incubate between 37°C and 42°C (Kellner et al. 2019). Modifications to Cas nucleases to increase their thermostability have allowed the integration of LAMP into one‐pot reactions (Nguyen et al. 2023), although this has yet to be seen in CRISPR‐eBx applications. After amplification, CRISPR‐Cas‐based detection is then evaluated by instrument‐assisted fluorescence quantification, qualitative assessment of UV‐inducible fluorescence (Blasko et al. 2024; Hoenig et al. 2023, 2024; Leugger et al. 2024; Pérez et al. 2024; Williams et al. 2019, 2021, 2023) or visualisation of linear indicators on lateral flow strips (Baerwald et al. 2020; Blasko and Phelps 2024; Huang et al. 2024; Leugger et al. 2025; Shashank et al. 2024; Yang et al. 2024; Figure 2). Although qualitative fluorescence, which requires only a dark box and ultraviolet light (Hoenig et al. 2023) and lateral flow strips (Baerwald et al. 2020), is the most cost‐efficient and field‐deployable option, research operations seeking quantitative sensitivity could utilise recently developed portable real‐time thermocyclers. Fluorescence data have enabled quantification of nucleic acid targets in both an RPA‐Cas13 (Gootenberg et al. 2018) and a LAMP‐Cas12 system (Li et al. 2019). Additionally, breakthroughs in human pathogen screening suggest that CRISPR‐Dx can yield highly sensitive detection without amplification, performing the nucleic acid detection step directly from crude RNA or DNA extracts (Ma et al. 2023). Early insights from these extraction‐free and amplification‐free assays hold great promise for using even simpler ‘one‐pot’ reactions to detect organisms and their gene expression directly from specimen tissues or swabs. This may be more difficult when working with eDNA samples in which target copy numbers are often low and thus pre‐amplification is required to increase sensitivity (Williams et al. 2019).

While some early CRISPR‐Dx rapid tests for human pathogens have employed Cas9 nucleases (Wu et al. 2024), studies in ecology and conservation are largely centered around the DNA‐targeting Cas12 nuclease (Blasko et al. 2024; D'Agnese et al. 2023; Hoenig et al. 2024; Kim et al. 2024; Pérez et al. 2024; Williams et al. 2019, 2021, 2023) and to a lesser extent the RNA‐targeting Cas13 (Baerwald et al. 2025; Durán‐Vinet et al. 2025; Nagarajan et al. 2024; Yang et al. 2024), which each display trans cleavage of non‐target nucleic acids upon recognition of the target molecule (Abudayyeh et al. 2016; Chen et al. 2018). Despite the ability of Cas13 to target and degrade RNA molecules, it has principally been used for DNA detection based on the original SHERLOCK methodology (Specific High‐sensitivity Enzymatic Reporter unLOCKing) (Gootenberg et al. 2017), which involves incorporation of a T7 promoter during pre‐amplification and the inclusion of a DNA‐to‐RNA transcription step prior to Cas13 cleavage (Nagarajan et al. 2024). However, Cas13's ability to detect RNA at attomolar sensitivity (1 × 10^−18^ M) indicates that it has great potential for eRNA monitoring (Yang et al. 2024), which can potentially provide higher temporal resolution than eDNA due to its faster degradation rate (Yates et al. 2021; Kagzi et al. 2022). This could be particularly useful for tracking migratory fish as well as assessing the environmental load of active stages of pathogens or the immune function of their hosts from eRNA. Furthermore, as Cas13 cleaves the target RNA only following the post‐amplification transcription step, it is more easily amenable to a ‘one pot’ reaction than Cas12 which may degrade target DNA prior to its necessary amplification when performed as a single reaction (Gootenberg et al. 2017; Li et al. 2024). This differentiation of amplification and detection steps across nucleic acid types (amplification of DNA, detection of RNA) enables Cas13 assays to be combined into a single reaction and thus may prove advantageous when moving towards simplified on‐site CRISPR‐eBx approaches. A ‘one pot’ reaction would significantly shorten assay times and limit the number of liquid volume transfers required, thus lessening the chances for cross‐contamination.

Importantly, several Cas nucleases, including Cas12 (Zetsche et al. 2015), are limited by the need for a short recognition sequence, known as the protospacer adjacent motif (PAM), to be present within the target nucleic acid. Different Cas nucleases are known to have different canonical PAM sites, for example, Cas12a has a T‐rich PAM (Zetsche et al. 2015) and Cas9 has a G‐rich PAM (Jinek et al. 2012). Furthermore, nuclease orthologs (the same nuclease from different species) may prefer differing PAMs and even show suboptimal recognition of non‐canonical PAM sites (Yamamo et al. 2017; Zhang et al. 2014, 2019). This necessity for the presence of a PAM site may limit gRNA design. On the other hand, some nucleases, such as Cas13, are PAM‐independent allowing more versatile gRNA placement (Cox et al. 2017). The discovery or synthesis of Cas nucleases with alternative PAM sites is increasing the overall applicability of CRISPR‐Cas methodologies and even allowing for versatile multiplex reactions that can discriminate several species from a single amplicon (Xu et al. 2022; Leugger et al. 2024).

In addition to PAM site requirements, specific assay design requires the location of mismatches with non‐target sequences to be considered. The seed sequence describes the region of the target molecule that requires the highest specificity for gRNA annealing. In PAM‐dependent nucleases, this commonly comprises several bases adjacent to the PAM site (Semenova et al. 2011). Mismatches between target and gRNA in this region reduce gRNA binding whereas several mismatches outside the seed sequence often do not prevent the activation of cleavage by the nuclease (Jost et al. 2020; Murugan et al. 2020). This mismatch tolerance outside of the seed region may prove beneficial for species identification in CRISPR‐eBx applications, whereby intraspecific variation might be present but not known about due to a lack of publicly available sequence data.

For single base resolution (i.e., CRISPR‐Dx assays that can discriminate single nucleotide polymorphisms), as would be desired for population‐wide genotyping or resistance tracking in disease ecology, some adjustments to gRNA design are necessary. Generally, single‐base resolution may be achieved by shortening the homologous section of the gRNA as far as possible to increase the ‘weighting’ of single base differences in guide‐target binding. However, targeting an overall shorter complementary sequence can increase the probability for off‐target activity which is particularly relevant in amplification‐free approaches. In the case of Cas13a, single guide‐target sequence mismatches often do not prevent activation of cleavage, while two mismatches in the seed sequence (bases 9–14 from 3′‐end of the gRNA) can suppress nuclease activity if consecutive (Abudayyeh et al. 2016; Liu et al. 2017; Tambe et al. 2018; Gao et al. 2021). To allow SNP detection using a Cas13 effector, mismatches can be intentionally incorporated into the synthetic gRNA at bases consecutive to the SNP, effectively mimicking two mismatches in the seed sequence and suppressing cleavage, and thus detection, of non‐target sequences (Mantena et al. 2025). For Cas12, target sequences that differ by only a single base in the seed sequence can already be discriminated, although the specificity strongly depends on the position of the SNP within the seed sequence (PAM‐proximal bases 1–6; Kim et al. 2016; Kleinstiver et al. 2016; Swarts et al. 2017). In the case of Cas9, likely the best characterised Cas nuclease to date, gRNA design has been studied extensively, showing that its activity is highly sequence‐dependent and that single‐base resolution can be achieved within the seed sequence (PAM‐proximal bases 1–10; Jinek et al. 2012; Cong et al. 2013; Fu et al. 2016). These findings suggest that similar properties may also exist in less studied Cas enzymes.

### 2.2 Enhanced Sequencing

Sequence selection is a common tool in molecular ecology to reduce sequencing costs and increase read depth by targeting only desired regions of a genome or desired species from metagenomic samples (Mamanova et al. 2010). CRISPR‐Cas technology has vast potential to enhance the specificity of sequencing libraries, either through the enrichment of target sequences or through the depletion of contaminant reads (Kardailsky et al. 2025).

There are two main approaches to a CRISPR‐capture strategy for target enrichment: use of a deficient Cas enzyme (i.e., a nuclease that is unable to cleave target nucleic acids) labelled with affinity tags for capture (Lee et al. 2019) or Cas nuclease cleavage of target molecules to create sticky ends that allow target‐specific adapter ligation, for example, FLASH (Finding Low Abundance Sequences by Hybridization; Quan et al. 2019). Enrichment is particularly interesting for eDNA applications because capture of species‐specific fragments can be performed prior to amplification and can thus help to overcome detection and quantification biases caused by nucleic acid amplification. Capture of nucleic acids directly from crude extracts is beneficial for more quantitative approaches, such as quantifying species‐specific chloroplast molecules (Littleford‐Colquhoun and Kartzinel 2024), or for detecting methylation rates for demographic estimates (Ruiz et al. 2025). Furthermore, CRISPR‐capture approaches are more versatile for long‐read sequencing, for example, ‘nCATs’ enrichment (nanopore Cas9‐targeted sequencing; Gilpatrick et al. 2020) and FUDGE (FUsion Detection from Gene Enrichment; Stangl et al. 2020) when compared to standard sequence capture approaches. Additionally, affinity‐tagged Cas enzymes and multiple gRNAs targeting short, conserved, genomic regions across the desired taxonomic group (e.g., fishes) may be able to enhance the recovery of unknown targets (e.g., recently arrived novel invasive species) that may be in low abundance in environmental samples. Overall, while previous studies have shown their efficacy in generating entire mitochondrial (Ramón‐Laca et al. 2023) and chloroplast genomes (Littleford‐Colquhoun and Kartzinel 2024), CRISPR‐capture remains underutilised in ecological contexts.

Another CRISPR‐Cas application for high‐throughput sequencing is the targeted depletion of undesired DNA from sequencing libraries using approaches such as DASH (Depletion of Abundant Sequences by Hybridisation; Gu et al. 2016). This has great potential in the field of eDNA metabarcoding whereby universal primers often amplify large quantities of non‐target DNA (e.g., human sequences amplified by vertebrate primers). Currently, the inclusion of PCR blocking primers is the most used method for suppressing non‐target sequences in metabarcoding (Calvignac‐Spencer et al. 2013; Polling et al. 2024). Unfortunately, these primers may have low blocking efficiencies caused by nucleotide mismatches with the sequence being blocked, or may block target sequences from amplification due to unintended complementarity between the primer and the target sequences. Instead, CRISPR‐Cas depletion uses highly specific gRNAs with active nucleases to digest these non‐target nucleic acids—either before or after amplification—and consequently increases target sequence coverage by preventing sequencing competition with undesired molecules (Kardailsky et al. 2025). This methodology has predominantly been used in the biotechnology field (Schultzhaus et al. 2021), though Owens et al. (2024) successfully used a modified DASH protocol to drastically reduce undesired host signal in 18S rRNA gene metabarcoding of host‐associated eukaryotes. However, Cas enzymes tend to display only single‐turnover nuclease activity on the target nucleic acid since each nuclease complex typically cleaves its target once and then becomes inactive rather than continuously processing multiple targets (Sternberg et al. 2014). Therefore, fine‐tuning may be necessary to ensure there is an adequate concentration of Cas to sufficiently deplete the non‐target molecules present in a sample.

### 2.3 Genome Editing

Genome editing with CRISPR‐Cas systems holds transformative potential for ecological and biodiversity research by enabling precise genetic manipulation to investigate mechanisms of adaptation, plasticity, and resilience in natural populations. There is substantial literature highlighting the promise of genome editing for conservation purposes (Johnson et al. 2016; Corlett 2017; Phelps et al. 2019; Yin et al. 2024). This includes using gene drives to suppress invasive species (Champer et al. 2016), increasing resilience against invasive diseases such as amphibians threatened by chytridiomycosis (Becker et al. 2021) or American chestnut trees threatened by chestnut blight (Newhouse et al. 2014), as well as increasing climate resilience in threatened species such as assisting heat tolerance in corals and their endosymbionts (Van Oppen et al. 2015). However, despite growing enthusiasm, these approaches remain largely theoretical due to numerous ecological, ethical, and regulatory challenges, including potential unintended effects on ecosystems (Akbari et al. 2015; Westra et al. 2016; Fernandez I Marti and Dodd 2018; Phelps et al. 2019). Such ecological effects may be hard to predict, irreversible, and transboundary, especially for systems designed to spread (e.g., gene drives). This complicates consent, sovereignty, and liability across jurisdictions and communities. A precautionary ethical framework, as suggested by Phelps et al. (2019), is therefore essential and should be grounded in rigorous ecological modeling, phased testing with clear exit criteria, and predesigned reliable containment measures coupled with robust post‐release monitoring before any conservation deployment is contemplated (Adams and Redford 2021).

At present, CRISPR‐based genome editing nevertheless offers strong potential for fundamental ecological research in controlled laboratory settings. Genome editing allows ecologists to dissect the genomic basis of evolutionary processes, such as resistance to emerging wildlife pathogens, without the immediate risks associated with releasing modified organisms into the wild (Fernandez I Marti and Dodd 2018; Phelps et al. 2019; Yin et al. 2024). Such studies can advise applied conservation genomics, guide conservation prioritisation, and support predictive models of species responses to environmental change.

## 3 Future Directions and Challenges of CRISPR‐Cas Applications

Despite the slow integration, CRISPR‐Cas technologies are being adapted for use in ecology and conservation and have already shown great promise for nucleic acid detection from swabs and environmental samples, as well as for enriching sequencing libraries for diet analysis and whole organelle sequencing. However, challenges requiring further research persist and need tackling if CRISPR‐Cas is truly to enhance ecological practices.

As we continue to better understand the underpinnings of gRNA sequence specificity—knowledge which is critical for sensitive and specific experimental design regardless of CRISPR‐Cas application—the distinction between single nucleotide polymorphisms is increasingly possible (Rabinowitz and Offen 2021). This paves the way for applications in population studies in real time, for instance in tracking alleles that underlie adaptation (Fernandez I Marti and Dodd 2018). The next steps for this rapidly growing field include experimentally engineering more resilient populations as well as optimising the quantification of nucleic acids and developing standard environmental detection assays for threatened species such as those in the IUCN Red List of Threatened Species (Phelps et al. 2019).

A general limitation of current CRISPR‐eBx assays is their predominantly singleplex nature (i.e., detecting one target sequence at a time). There is thus a methodological need to develop more scalable multiplex assays or those that enable detection of more than one target species in a single workflow (even if not multiplexed in the true sense), such as the ‘ampliscanning’ approach of Leugger et al. (2024). This method relies on PCR amplification (using metabarcoding primers) of a broad taxonomic group, followed by the application of various discriminatory gRNAs to detect multiple species from a single amplicon. Multi‐species assays can be multiplexed by using several nucleases with different nucleotide cutting preferences and reporter molecules (Gootenberg et al. 2018) as well as through microfluidic spatial separation of Cas nucleases and species‐specific gRNA sequences (Shao et al. 2019). High throughput CRISPR‐Dx approaches, combined with microfluidic technology, have been used to monitor human viruses such as HPV subtypes (Xu et al. 2022) and respiratory viruses including SARS‐CoV‐2 (Welch et al. 2022), but have yet to be utilised in an ecological context. Development in this way would significantly enhance the throughput and cost‐efficiency of CRISPR‐Cas based species detection. Furthermore, CRISPR‐Dx cannot detect unknown targets de novo such as in community metabarcoding or screening for novel pathogens. Recent advances in isothermal approaches for metagenetic amplification (Plewnia et al. 2025) may help circumvent this and enable on‐site community analysis, complementing field‐deployable CRISPR‐eBx applications. Unlike CRISPR‐Dx, CRISPR‐enhanced high‐throughput sequencing offers new opportunities to advance de novo detection. Future approaches could include highly multiplexed CRISPR‐capture workflows that enrich thousands of loci across diverse taxa from a single environmental sample, enabling real‐time ecosystem‐level genomic monitoring. Combined with long‐read sequencing, such methods may allow the reconstruction of complete organellar or even nuclear genomes from rare or degraded samples, providing critical data for conservation genomics and species discovery.

In the context of CRISPR‐eBx, nearly all currently available assays have been developed and tested in temperate regions, primarily focusing on northern hemisphere biodiversity. Studies have been dominated by aquatic species detection with little to no research showing the applicability of the method in terrestrial or aerial environments. This follows a broader trend in eDNA studies whereby there is a clear bias towards monitoring in the global North (Schenekar 2023), and in particular freshwater ecosystems and fish species (Sahu et al. 2025). This geographic and environmental bias underscores a pressing need to adapt CRISPR‐eBx to better suit tropical and understudied ecosystems, which harbour the vast majority of global biodiversity. Expanding assay design and validation efforts to encompass these regions will be critical for affordable, accessible and globally relevant biodiversity monitoring.

Future work should integrate FAIR principles (Findable, Accessible, Interoperable, Reusable) (Takahashi et al. 2025) and prioritise transparent reporting of reaction conditions to support responsible innovation. This is especially vital for nucleic acid detection workflows, where reproducibility remains a key bottleneck. Similar to other molecular techniques, detailed methodological documentation is essential to enable cross‐study comparability. Moreover, future CRISPR‐eBx assays should be totally field‐deployable to increase their accessibility. This requires the use of on‐site DNA extraction (Blasko and Phelps 2024; Blasko et al. 2024; Hoenig et al. 2023, 2024; Yang et al. 2024) or extraction‐free assays (Baerwald et al. 2020, 2025) and improvements in visualisation methods including simplified fluorescence instrumentation (Williams et al. 2025) or enhanced lateral flow strips. Such developments will further increase the capability of CRISPR‐eBx approaches for rapid, on‐site monitoring. Additionally, neural networks have been developed for both Cas12 (Huang et al. 2024) and Cas13 (Metsky et al. 2022; Durán‐Vinet et al. 2025) to improve the design of CRISPR assays, regardless of their application. These tools aim to rapidly identify specific gRNA sequences that show minimal off‐target effects. Such tools would simplify the design of CRISPR‐Cas based assays making their application more accessible to researchers and practitioners. Ultimately, more research is needed to delimit and extend the current applicability of CRISPR‐Cas in ecology. We urge ecologists to make further use of this powerful and versatile technology to unravel, monitor and preserve earth's biodiversity.

## Author Contributions

A.P., B.D.H. and M.A.W. conceptualised this work and wrote the first draft. Revisions to the manuscript were performed by A.P. and M.A.W. All authors edited the manuscript and approved the final version.

## Funding

This work was supported by Natural Environment Research Council, NE/S010270/1; University of Warwick.

## Conflicts of Interest

The authors declare no conflicts of interest.

## Data Availability

The authors have nothing to report.
